# Low-Dose X-Ray Irradiation Promotes Osteoblast Proliferation, Differentiation and Fracture Healing

**DOI:** 10.1371/journal.pone.0104016

**Published:** 2014-08-04

**Authors:** Ming Chen, Qun Huang, Wei Xu, Chang She, Zong-Gang Xie, Yong-Tao Mao, Qi-Rong Dong, Ming Ling

**Affiliations:** 1 Department of Orthopedics, Shaanxi Provincial Peoples Hospital, Xi'an, Shaanxi, China; 2 Department of Orthopedics, Second Affiliated Hospital of Soochow University, Suzhou, Jiangsu, China; University of Ulm, Germany

## Abstract

Great controversy exists regarding the biologic responses of osteoblasts to X-ray irradiation, and the mechanisms are poorly understood. In this study, the biological effects of low-dose radiation on stimulating osteoblast proliferation, differentiation and fracture healing were identified using in vitro cell culture and in vivo animal studies. First, low-dose (0.5 Gy) X-ray irradiation induced the cell viability and proliferation of MC3T3-E1 cells. However, high-dose (5 Gy) X-ray irradiation inhibited the viability and proliferation of osteoblasts. In addition, dynamic variations in osteoblast differentiation markers, including type I collagen, alkaline phosphatase, Runx2, Osterix and osteocalcin, were observed after both low-dose and high-dose irradiation by Western blot analysis. Second, fracture healing was evaluated via histology and gene expression after single-dose X-ray irradiation, and low-dose X-ray irradiation accelerates fracture healing of closed femoral fractures in rats. In low-dose X-ray irradiated fractures, an increase in proliferating cell nuclear antigen (PCNA)-positive cells, cartilage formation and fracture calluses was observed. In addition, we observed more rapid completion of endochondral and intramembranous ossification, which was accompanied by altered expression of genes involved in bone remodeling and fracture callus mineralization. Although the expression level of several osteoblast differentiation genes was increased in the fracture calluses of high-dose irradiated rats, the callus formation and fracture union were delayed compared with the control and low-dose irradiated fractures. These results reveal beneficial effects of low-dose irradiation, including the stimulation of osteoblast proliferation, differentiation and fracture healing, and highlight its potential translational application in novel therapies against bone-related diseases.

## Introduction

Bone development and homeostasis are maintained through the balance between bone-forming osteoblasts and bone-resorbing osteoclasts. Osteoblasts are the chief bone-making cells that are responsible for the production of bone extracellular matrix during bone remodeling or healing [1]. During the process of bone formation, osteoblasts must proliferate, differentiate and induce mineralization of the bone extracellular matrix. A variety of genes in osteoblasts, including fluctuations in type I collagen (Col1), alkaline phosphatase (ALP), osteopontin (OPN), osteonectin (ON), osteocalcin (OCN) and others, undergo characteristic sequential changes in phenotypic gene expression during this series of events [2].

It is well known that high-dose irradiation delivers deleterious effects to bone tissue, including osteoradionecrosis, sclerosis, loss of bone mass and bone fracture, in a dose- and time-dependent manner [3]–[6]. Damage to osteoblasts and osteocytes is thought to be a primary contributor to the reduced bone mineral density that is observed following irradiation. Previous studies have suggested that irradiation can impair bone formation by impeding osteoblast proliferation and differentiation, inducing cell-cycle arrest, reducing collagen production and increasing the sensitivity to apoptotic agents [7], [8]. However, the effects of low-dose irradiation, especially at levels less than 1Gy, on bone responses and healing have rarely been described in the literature. Orthopedic patients are more often subjected to radiation exposure, such as radiography, computed tomography or fluoroscopy during surgery, where the exposure to ionizing radiation is typically at low-dose levels (<1 Gy) [9], [10]. In addition, several studies have revealed the detrimental effects of low-dose irradiation (<1 Gy) through various molecular mechanisms, including increases in reactive oxygen species formation, DNA double-strand breaks and chromosomal breakage [11]–[13]. Conversely, other studies have reported contrasting results concerning low-dose irradiation. For example, Li *et al*. reported that 75 mGy X-ray irradiation induced maximal stimulation of bone marrow hematopoietic progenitor cell proliferation and a significant increase in the mobilization of these cells to the peripheral blood post-irradiation [14]. In addition, Liang *et al*. reported that the proliferative capacity of bone marrow-derived mesenchymal stem cells (BM-MSCs) exposed to low-dose X-rays was significantly enhanced compared with the non-irradiated control group [15]. Nevertheless, the biological response of osteoblast lineage cells to low-dose X-ray irradiation remains controversial. Recently, the study performed by Pramojanee *et al.* showed that 1.5 mGy X-ray irradiation decreased oxidative stress in osteoblasts and did not alter cell viability, cellular proliferation or cellular apoptosis [11]. A previous study demonstrated that 2 Gy X-ray ionizing radiation induced time-dependent cell cycle arrest and had no significant effects on osteoblast proliferation and differentiation in an osteoblastic cell line [16], [17]. However, Park *et al.* [18] reported that 2 Gy X-ray irradiation not only increased differentiation and mineralization of the cells but also upregulated the expression of ALP, Col1, OPN and OCN in the early stage of differentiation.

It is generally considered that sequential proliferation and differentiation of osteoblasts is indispensable for bone remodeling and healing. Our preliminary studies surprisingly demonstrated that 1 Gy X-ray irradiation promoted callus formation and mineralization in a rat model [19], and this type of radiation was also shown to have different effects on the proliferation and differentiation of osteoblasts in vitro (0.5–1 Gy) [20]. However, the underlying mechanism has not been evaluated in osteoblasts exposed to low-dose irradiation. In this study, we investigated the mechanism by which low-dose X-ray irradiation (0.5 Gy) influences the proliferation and differentiation of osteoblasts and promotes fracture healing. Osteogenic gene expression patterns were evaluated to explore the possible mechanisms involved in irradiation-stimulated osteoblast differentiation *in vivo* and *in vitro*. Our findings provide a greater understanding of the biological responses of osteoblasts exposed to low-dose X-ray irradiation and highlight the potential positive effects of this treatment.

## Methods

### Cell culture and irradiation

MC3T3-E1 subclone 14 cells were purchased from the Chinese Academy of Sciences Cell Bank. The same procedures, such as seeding time, cell numbers and replacing osteogenic medium, were performed during our study. Cells were cultured in α-MEM (HyClone, USA) containing 10% fetal bovine serum (Gibco, USA), 100 U/ml penicillin and 100 µg/ml streptomycin. In the osteoblast differentiation experiments, the culture media was replaced with osteogenic medium supplemented with 50 µg/ml ascorbic acid (Sigma-Aldrich, USA) and 10 mM β-glycerophosphate (Sigma-Aldrich, USA) after the cells had achieved 70–80% confluence. The cells were exposed to a single 0.5 Gy (low-dose) or 5 Gy (high-dose) dose of X-ray irradiation at a rate of 200 cGy/min using a medical linear accelerator with a 6 MV radiation source (Siemens Primus, Concord, CA, USA). All irradiations were performed at room temperature, and the control samples were treated similarly but were not irradiated. The time at irradiation was termed day 0. The cells were maintained in a 5% CO_2_ atmosphere at 37°C, and the medium was exchanged every 3 days.

### Fracture model establishment and irradiation

All animal studies were performed in strict accordance with the recommendations in the Guide for the Care and Use of Laboratory Animals of the National Institutes of Health. The protocol was approved by the Committee on the Ethics of Animal Experiments of the Second Affiliated Hospital of Soochow University. All surgeries were performed under chloral hydrate anesthesia, and all efforts were made to minimize suffering.

Male Sprague-Dawley rats (body weight 220±20 g) were obtained from the Animal Unit Center of Soochow University. A total of 96 rats were used in our *in vivo* studies. The animals were acclimated for 7 days prior to the experiments and were administered food and water ad libitum. Closed femur fractures were created as described previously [19]. Briefly, the rats were anesthetized with 3.6% chloral hydrate (360 mg/kg, intraperitoneal injection). A lateral parapatellar knee incision was made to expose the distal femoral condyles. A 1.2-mm diameter K-wire was inserted into the length of the medullary canal of the femur from the trochlear groove until the wire exited through the greater trochanter and the skin. The distal end was positioned deeply into the articular surface of the knee, and a mid-diaphyseal fracture was created via three-point bending with a custom-made guillotine-like fracture apparatus. Clear oblique fracture lines or comminuted fractures were excluded based on radiography performed after the surgery. Then, the established fracture animal models were randomized into the irradiation and control groups. The animals in the irradiation group were administered a single dose (0.5 or 5 Gy) of X-ray irradiation locally to the operated thigh using a linear accelerator with a 6 MV radiation source. Control rats were treated in the same manner as the irradiation groups; however, these rates were not exposed to X-ray irradiation.

### CCK-8 assay for cell viability

MC3T3-E1 cells after irradiation were seeded in 96-well plates at a density of 3000 cells/well and cultured in an incubator for 1- to 7-day periods. The Cell Counting Kit-8 (CCK-8; Dojindo, Japan) was employed to quantitatively evaluate cell viability. Briefly, α-MEM (100 µl) with 10 µl CCK-8 was added to each pre-cultured well, and the plates were then incubated for 2 h at 37°C. The absorbance was determined at a wavelength of 450 nm using a TECAN Infinite M200 microplate reader (Tecan Group Ltd., Switzerland).

### Bromodeoxyuridine (BrdU) incorporation for cell proliferation

The cells were plated and treated as described above for 2, 4 or 6 days. A BrdU ELISA kit (Roche, Germany) was used to determine cell proliferation according to the manufacturer's instructions. First, BrdU was added to the medium, and the plates were incubated for 2 h at 37°C. Then, the cells were fixed, and the DNA was denatured for 30 min at room temperature after removal of the culture medium. The anti-BrdU antibody was then added to bind to the BrdU that had been incorporated into the newly synthesized cellular DNA. Finally, the immune complexes were detected by the subsequent substrate reaction, and the absorbance of each well was measured using a TECAN Infinite M200 microplate reader at 370 nm (492 nm reference wavelength).

### Flow cytometric analysis

The cell cycle progression of MC3T3-E1 cells was analyzed by flow cytometry. After irradiation, the cells were incubated for 3 days. The harvested cells were washed twice with PBS, fixed with ice-cold 70% ethanol and stored at 4°C overnight. The fixed cells were then washed with PBS again and incubated in 100 µl RNase A/PBS (200 µg/ml) at 37°C for 30 min. Intracellular DNA was labeled with 300 µl propidium iodide (PI, 10 µg/ml) at 4°C for 15 min, and the stained cells were analyzed on a flow cytometer for relative DNA content based on increased red fluorescence. The data were presented as percentages of the total population. Greater than 1×10^4^ cells were analyzed per sample, and 3 independent experiments were conducted.

### Alizarin Red staining

The degree of mineralization in MC3T3-E1 cells was determined in 24-well plates by Alizarin Red staining. Irradiated osteoblasts were fixed for 1 h in ice cold 70% ethanol 21 days after irradiation. The cells were then washed thoroughly with deionized H_2_O, and mineralization was detected with 40 mM Alizarin red (pH 4.2) for 15 min. Following staining, cells were washed thoroughly with deionized H_2_O to remove non-specific Alizarin red staining. After air-drying, the cell culture plates were evaluated by light microscopy.

### Western blot analysis

The cells were homogenized in RIPA lysis buffer (Beyotime, China) using a sonic dismembrator containing protease and phosphatase inhibitors (Roche, Switzerland). Freshly isolated cell homogenates were centrifuged for 10 min at 12,000×g and 4°C. The supernatant was collected, and the protein concentration was determined using a bicinchoninic acid (BCA) protein assay kit (Beyotime, China). All samples were then diluted to an equal concentration in PBS and boiled for 5 min. Equal amounts of protein (30 µg) were subjected to 8–15% SDS-PAGE and then transferred onto polyvinylidene fluoride (PVDF) membranes. After blocking with 5% w/v non-fat dry milk for 1 h at room temperature, the membranes were incubated overnight with primary antibodies against β-actin (ab133626, Abcam, USA; dilution 1∶3000), Col1α (ab34710, Abcam, USA; dilution 1∶5000), ALP (ab95462, Abcam, USA; dilution 1∶2000), OCN (AB10911, Millipore, USA; dilution 1∶1000), Runx2 (sc-10758, Santa Cruz, USA; dilution 1∶500) and Osterix (ab22552, Abcam, USA; dilution 1∶2000) at 4°C with gentle shaking. After washing thrice in PBST, the blots were incubated with a 1∶2000 dilution of goat anti-rabbit IgG horseradish peroxidase (HRP)-conjugated secondary antibody (ab97200, Abcam, USA) for 2 h at room temperature. The blots were washed thrice and visualized using a chemiluminescence kit (BioInd, Israel). Images were then captured with a chemiluminescence imaging system (Syegene, UK). Given that protein levels differ in relative abundance, exposure times or image intensities were varied to produce linear stain-to-background ratios.

### Real-time quantitative PCR analysis

Rats were euthanized at 7, 14, 21 and 28 days after irradiation (4 rats per group). The fracture callus and 1–2 mm of the normal bone margin were carefully excised from the soft tissue using a scalpel. The samples were frozen in liquid N_2_, pulverized using a nitrogen-cooled mortar and pestle apparatus and purified for total RNA using the Trizol system (Invitrogen, USA) according to the manufacturer's instructions. First-strand cDNA was obtained from the total RNA (1 µg) using RevertAid M-MuLV reverse transcriptase and oligo (dT) primer (Fermentas, Lithuania). Real-time PCR was performed in a total volume of 20 µl, which consisted of 1 µl of cDNA (500 ng), 1 µl of gene-specific 10 µM PCR primer-pair stock and 10 µl of SsoFast EvaGreen Mix (Bio-Rad, USA), using the Bio-Rad CFX96 system according to the manufacturer's protocol. The primer sequences for β-actin, Col1a1, Col2α1, OCN, ALP, Runx2 and Osterix are presented in Table 1. The PCR conditions consisted of an initial step at 95°C for 30 s to activate the DNA polymerase followed by 40 cycles of 5 s at 95°C and 5 s at 60°C, which were subsequently followed by the melting curve test. The relative amount of mRNA expression normalized to β-actin was expressed as a fold change, which was calculated using the comparative Ct (2^−ΔΔCt^) method using the control group as a reference with 2^−ΔΔCt^ = 1. Each sample was assayed in triplicate, and each experiment was independently repeated thrice.

**Table 1:** List of Oligonucleotide Primer Sequences for Real-Time PCR.

Gene	Primer Sequences
β-actin	Forward: 5′- GAGAGGGAA ATCGTGCGTGAC-3′
	Reverser: 5′- CATCTGCTGGAAGGTGGACA-3′
Col1α1	Forward: 5′-AAGGCCCACGGGGACCTGTT-3′
	Reverser: 5′-GGGCCAGGCACGGAA ACTCC-3′
Col2α1	Forward: 5′-CTACGGCGACGGCAACCTGG-3′
	Reverser: 5′-TGCCCTCGGCCCTCATCTCC-3′
OCN	Forward: 5′-GCGCATCTATGGCACCACCGT-3′
	Reverser: 5′-TTTGGA GCAGCTGTGCCGTCC-3′
ALP	Forward: 5′-GCCCAGGCA ACCTCGAGCAG-3′
	Reverser: 5′-TCCGACCCACGGAGGGTTCC-3′
Runx2	Forward: 5′-TCCAGGAGGACAGCAAGGAGGC-3′
	Reverser: 5′-TCGGTTGGTCTCGGTGGCTGG-3′
Osterix	Forward: 5′- AGAAGATCCCTCCCAGCGCCC-3′
	Reverser: 5′- GGGTTGGCTGTCCCGTCTCC-3′
OPG	Forward: 5′-ATCGGCCACGCGAACCTCAC-3′
	Reverser: 5′-GCTGCTCGCTGGGTTTGCAG-3′
RANKL	Forward: 5′-GGGCCA ACCGAGACTACGGC-3′
	Reverser: 5′-GGATGCAGCGGACCCTCGTG-3′

### Histology and analysis

The fractured femurs were harvested on days 7, 14, 21 and 28 post-irradiation (4 rats per group). Excess muscle and soft tissue were excised and fractured femurs were assessed by radiography. Four specimens from each group were fixed in 10% neutral buffered formalin. The specimens were decalcified for 4–5 weeks in 10% EDTA (pH 7.2), embedded in paraffin and sectioned at a thickness of 3 µm. The sections were cut through the long axis of each femur in the sagittal plane and stained with Masson's trichrome stain and TRAP stain (three levels per animal). Osteoclast counts were obtained from the number of TRAP-positive cells. The total callus area, total cartilage area and total woven bone area were quantified using Image Pro Plus 6.0 software as described previously [21].

Immunohistochemistry was also performed on these sections using a previously described method [22]. The sections were mounted on poly-L-lysine-coated glass slides and then air-dried overnight at room temperature. The sections were then deparaffinized with xylene and hydrated with serial concentrations of 100, 95, 80 and 70% alcohol. Endogenous peroxidase activity was blocked with 0.5% hydrogen peroxide in methanol for 60 min. Following a Tris-buffered saline (TBS)/0.1% bovine serum albumin (BSA) wash, hyaluronidase treatment (hyaluronidase 1 mg/ml in sodium acetate buffer, pH 5.5, 0.85% NaCl) was performed for 30 min at 37°C. After washing with TBS/0.1% BSA, the sections were incubated with blocking solutions (fresh 10 ml of TBS/0.5% BSA containing 130 µl of normal horse serum) at room temperature for 15 min. The sections were then incubated with the proliferating cell nuclear antigen (PCNA) monoclonal antibody (PC10; sc-56, Santa Cruz, USA) and OCN polyclonal antibody (AB10911, Millipore, USA) at a 1∶100 dilution. A subsequent reaction was performed using a Vectastain avidin/biotin-peroxidase complex (ABC) kit. The sections were reacted with diaminobenzidine (DAB) solution and were then counterstained with methyl green. We identified the cell type in the histological analyses based on tissue types, callus areas and cellular morphology [23]. Briefly, in the early stages after fracture, the calluses were mainly composed of soft tissue, including cell debris and mesenchymal cells, which exhibited a high proliferation activity. Chondrocytes were detected in the cartilage island. The premature cells were located in the margin of the cartilage adjacent to the soft tissue and were small and round in shape. The hypertrophic or mature chondrocytes were located in the center of the cartilage tissue or adjoined to the woven bone and displayed vacuole morphology. The vessel located in the soft tissue presented a cavity structure, which was encircled by flat endothelial cells.

### Statistical analysis

Statistical analyses were performed using SPSS version 17.0 (IBM SPSS, USA). All data are expressed as the means ± SD. One-way or two-way analysis of variance (ANOVA) and post-hoc multiple comparisons were used for statistical analyses. A difference was considered significant if the *p*-value was <0.05.

## Results

### Low-dose X-ray irradiation increases the cell viability and proliferation of osteoblastic MC3T3-E1 cells

**Figure 1:**
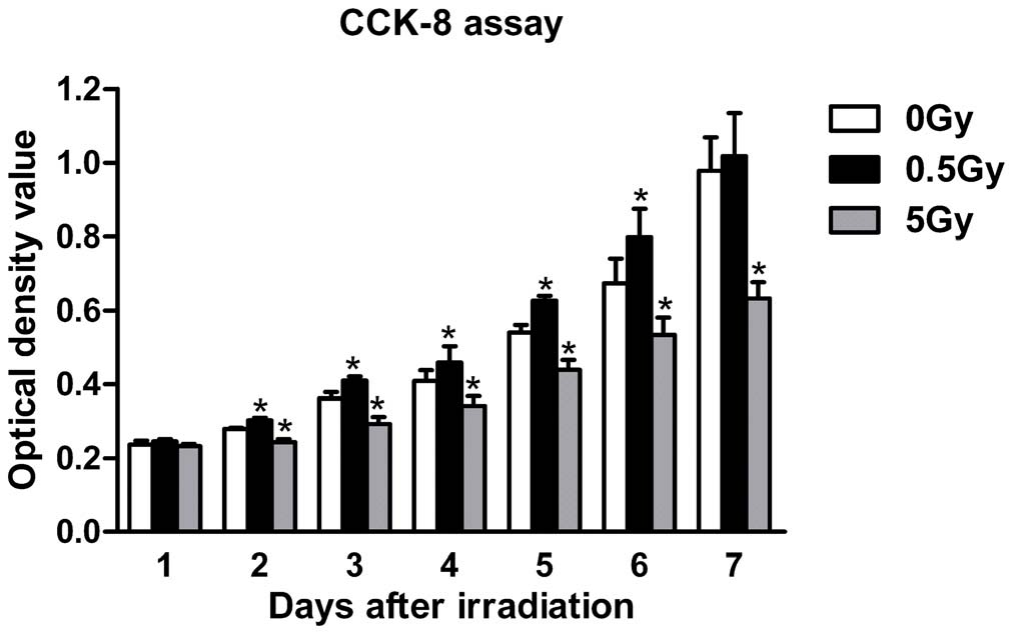
MC3T3-E1 cell viability as determined by the CCK-8 assay. Compared with the control cells (0 Gy), single low-dose (0.5 Gy) irradiation significantly increased cell viability from days 2 to 6, whereas high-dose (5 Gy) irradiation significantly reduced cell viability from days 2 to 7. *p<0.05 *vs.* the control group.

**Figure 2:**
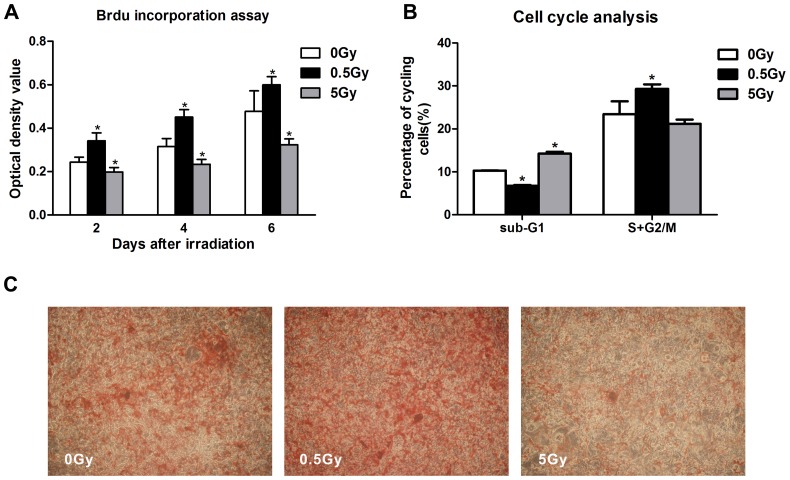
MC3T3-E1 cell proliferation and mineralization after irradiation. Low-dose (0.5 Gy) irradiation significantly promoted cell proliferation from days 2 to 6 compared with control cells (0 Gy), whereas high-dose (5 Gy) irradiation inhibited cell proliferation (A). Low-dose (0.5 Gy) irradiation resulted in an approximate 6.75% decrease in the percentage of cells in the sub-G1 phase, whereas high-dose (5 Gy) irradiation elicited an approximate 14.26% increase in the percentage of cells in the sub-G1 phase (B). Low-dose (0.5 Gy) irradiation accelerated osteoblasts mineralization 21 days after irradiation, and high-dose (5 Gy) irradiation decreased alizarin red staining (C). *p<0.05 *vs.* the control group.

We performed cell viability CCK-8 assays at days 1 through 7 following irradiation. As shown in Fig. 1, no statistically significant differences in CCK-8 activity in MC3T3-E1 cells were noted between the individually irradiated groups and the non-irradiated controls at day 1 post-irradiation. However, cells irradiated with a single, low dose (0.5 Gy) exhibited an increased in cell viability at days 2 to 6 (p<0.05), whereas high-dose (5 Gy) X-ray irradiation significantly reduced cell viability at days 2 to 7 (p<0.05). In addition, 0.5 Gy of irradiation exhibited no significant effect by day 7, and this result might be attributed to the notion that MC3T3-E1 cell growth had plateaued in both the control and the low-dose irradiation cultures. To determine whether this variation in cell viability was due to cell proliferation, BrdU incorporation was measured after irradiation. The results indicated that low-dose (0.5 Gy) X-ray irradiation promoted cell proliferation on days 2, 4 and 6 post-irradiation compared with the 0 Gy control (p<0.05), whereas high-dose (5 Gy) irradiation inhibited MC3T3-E1 cell proliferation (Fig. 2A). Cell cycle analysis by flow cytometry revealed that single, low-dose (0.5 Gy) X-ray irradiation induced a decrease in the proportion of cells at the sub-G1 phase, which indicates active apoptosis (6.75%±0.21%, p<0.05 *vs.* control), whereas increasing levels of cell death by apoptosis were observed in the high-dose irradiation group (14.26%±0.41%, p<0.05 *vs.* control). Moreover, our results indicated that low-dose irradiation caused a 5.85% increase in the S+G2/M phases of the cell cycle in osteoblasts compared with control (p<0.05), whereas high-dose irradiation had no effect (Fig. 2B). These results indicated that low-dose X-ray irradiation potentially promotes osteoblast survival and renewal.

### Low-dose X-ray irradiation promotes osteoblasts differentiation and mineralization in *vitro*

**Figure 3:**
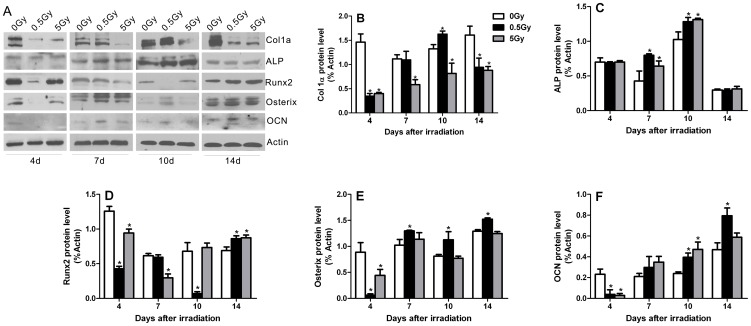
The effects of irradiation on the expression of osteoblast differentiation markers *in vitro*. The expression of Col1α, ALP, Runx2, Osterix and OCN proteins was assessed by Western blot on post-irradiation days 4, 7, 10 and 14 (A). The Col1α (B), ALP (C), Runx2 (D), Osterix (E) and OCN (F) protein levels were normalized to β-actin expression. *p<0.05 *vs.* the control group.

After X-ray irradiation, MC3T3-E1 cells were further cultured in osteogenic medium, and the expression levels of proteins associated with osteoblast differentiation were examined by Western blot at 4, 7, 10 and 14 days after irradiation. The statistical analysis histograms are presented in Fig. 3A–F. Osteoblast differentiation markers, such as OCN, Runx2 and Osterix, were inhibited temporarily at 4 days post-irradiation in both the low- and high-dose groups, with the levels of Runx2 and Osterix demonstrating the most prominent decreases in the low-dose group (Fig. 3A, D–F). The levels of Col1α were decreased at 4 and 14 days but were elevated at 10 days post-irradiation in the low-dose group, whereas a continual decrease in Col1α levels was observed in the high-dose group from days 4 to 14 after irradiation (Fig. 3A, B). Irradiation-stimulated increases in alkaline ALP levels occurred at 7 and 10 days after 0.5 Gy and 5 Gy X-ray irradiation (Fig. 3A, C). In addition, both the 0.5 Gy and 5 Gy radiation doses led to significant increases in OCN levels at 10 days, with the increases in the 0.5 Gy group continuing through 14 days post-irradiation (Fig. 3A, F). Decreased Runx2 levels were detected in the high-dose group at 7 days and in the low-dose group at 10 days; however, both low- and high-dose irradiation stimulated increases in Runx2 protein levels at 14 days after irradiation (Fig. 3A, D). In addition, the expression of Osterix in the low-dose group was significantly increased at 7, 10 and 14 days compared with the non-irradiated control group (Fig. 3A, E). These findings indicate that X-ray irradiation induced dynamic variations in the differentiation of pre-osteoblastic cells. Although we observed temporary reductions in osteogenic markers of early osteoblast differentiation after single-dose X-ray irradiation, dramatic increases in the related differentiation markers occurred at the medium differentiation. In addition, Alizarin Red staining demonstrated that low-dose irradiation promoted mineralization in MC3T3-E1 cells. For cells irradiated with high-dose irradiation, the mineralized bone nodules decreased in number (Fig. 2C). Together, low-dose X-ray irradiation promoted osteoblasts differentiation and mineralization in *vitro*.

### Low-dose X-ray irradiation accelerates endochondral and intramembranous ossification in fracture healing

**Figure 4:**
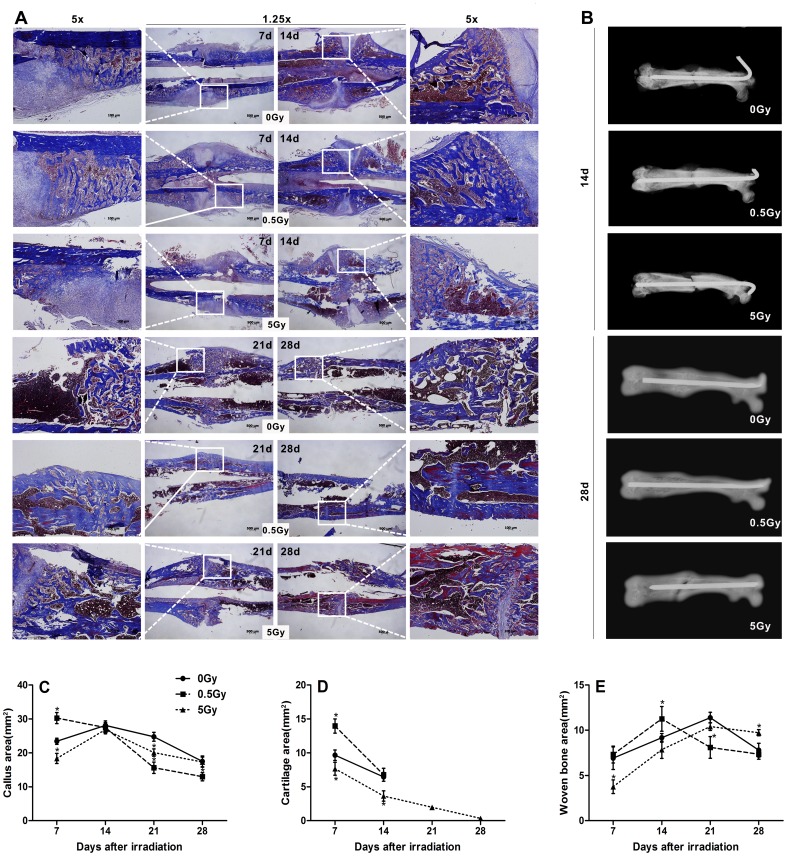
The effects of irradiation on fracture healing. Fractured femurs were harvested and immediately assessed by radiography. Representative histologies of fractured femurs stained with Masson's trichrome staining is depicted at 7, 14, 21 and 28 days after X-ray irradiation; accelerated remodeling on day 21 in fractures irradiated with low-dose X-ray irradiation was evident comparing the contracted calluses to the broad calluses that remained in the control fractures, whereas non-union on day 28 was observed in fractures that received high-dose X-ray irradiation (A). Radiographs demonstrated the accelerated callus mineralization and fracture repair (B). Histomorphometric measurements (total callus area, cartilage area, woven bone area) were performed, and the results are presented as a diagram (C, D, E). n = 4 rats/group, *p<0.05 *vs.* the control group.

A rat femur fracture model was employed to study fracture healing after irradiation. Fracture calluses from irradiated and non-irradiated rats, which were age- and sex-matched, were examined by radiography assessment and Masson's trichrome staining at days 7, 14, 21 and 28 following irradiation (Fig. 4). The radiographs indicate that 0.5 Gy X-ray irradiation accelerated callus mineralization and fractures repair, whereas 5 Gy X-ray irradiation delayed fracture healing (Fig. 4B). Histology sections of the calluses confirmed accelerated fracture healing and remodeling in low-dose (0.5 Gy) irradiated rats compared with the non-irradiated control group. However, the fractures in high-dose (5 Gy) animals demonstrated delayed healing. On day 7 following irradiation, low-dose irradiated rats presented increased callus areas and cartilage formation compared with non-irradiated rats. In contrast, decreased callus areas and less bone and cartilage formation were detected in high-dose irradiation rats (Fig. 4A, C–D). By day 14, the calluses in the high-dose group were also composed of less cartilage and woven bone area compared with the control group (Fig. 4A, D, E). On days 21 to 28, fractures in the low-dose irradiated and control rats were healed, and cartilage tissue was essentially absent in the calluses. However, healing was incomplete in the fractures of high-dose irradiated rats where a central area of cartilage tissue persisted (Fig. 4A, D). In addition to the accelerated osteogenesis, the fractures in the low-dose group underwent more rapid remodeling. In particular, low-dose irradiated rats exhibited abundant woven bone formation that peaked at 14 days; this event occurred earlier than the bone formation observed in the non-irradiated group, in which peak bone area was measured at 21 days. Subsequently, the bone areas of both groups progressively decreased (Fig. 4A, E). Beginning on day 21, low-dose-irradiated rats demonstrated decreased callus area, and most of the initial woven bone within the calluses had been remodeled with lamellar bone, which persisted through 28 days post-irradiation (Fig. 4A, C). By 28 days, the fractures in the low-dose irradiated rats had essentially undergone complete remodeling, and the femurs exhibited a similar morphology to non-fractured bone. In the control group, extensive remodeling was not observed until 28 days post-irradiation. Moreover, abnormal mineralization and remodeling were involved in fracture healing after high-dose irradiation. Fracture calluses in high-dose irradiated rats remained enlarged and contained areas of woven bone where greater amounts of mature collagen (red stained) were deposited at 28 days (Fig. 4A).

**Figure 5:**
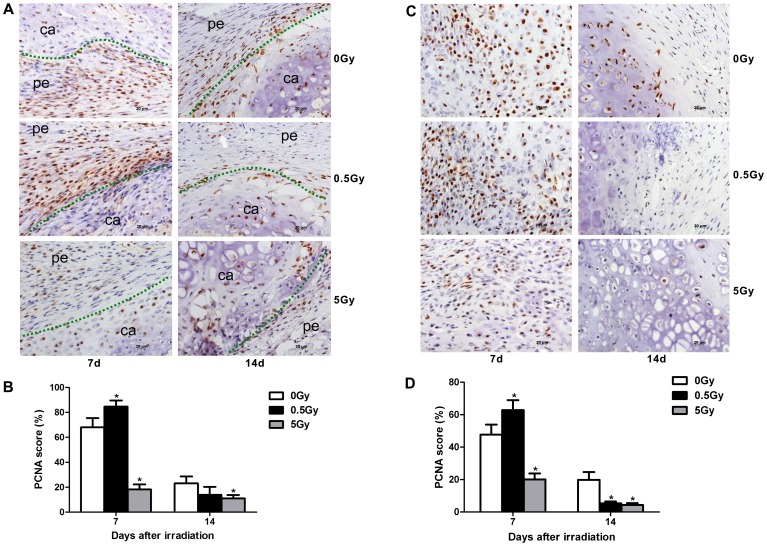
Cell proliferation during intramembranous osteogenesis and chondrogenesis. PCNA immunostaining was performed at 7 and 14 days after X-ray irradiation. Low-dose X-ray irradiation promoted the proliferation of periosteum cells and chondrocytes at day 7 after irradiation in fracture sites, whereas high-dose X-ray irradiation inhibited the proliferation of these cells at both days 7 and 14 post-irradiation (A, C). Quantification of cell proliferation in each group (B, D). The green dot represents the demarcation between the soft tissue and cartilage, and the white arrow denotes a vessel. pe: periosteum, ca: cartilage, n = 4 rats/group,*p<0.05 *vs.* the control group.

**Figure 6:**
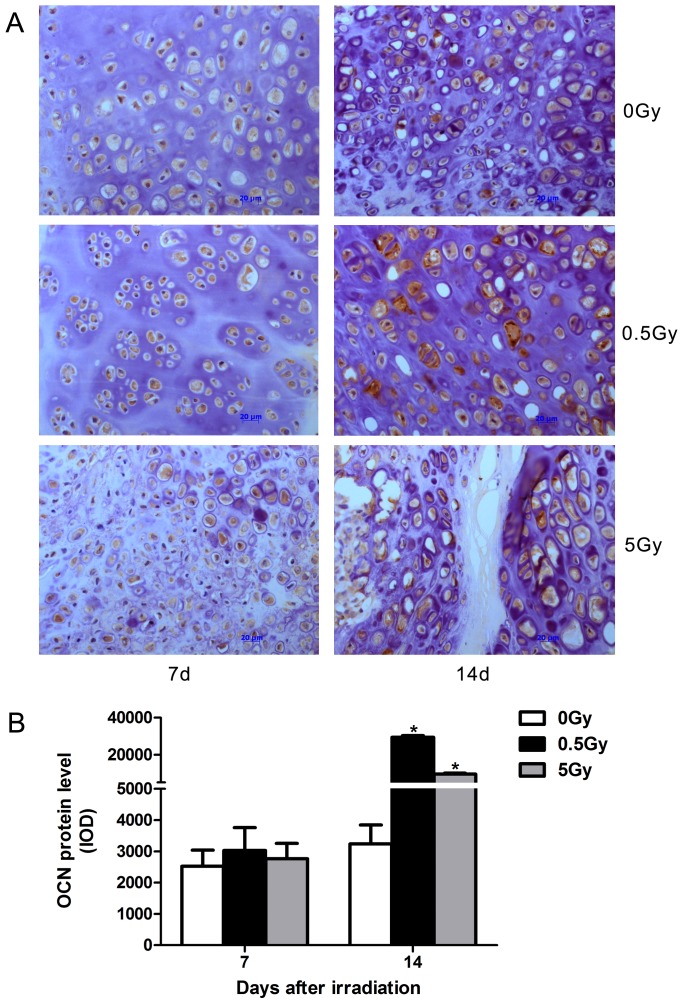
Irradiation increased OCN-positive cells in calluses on day 14 after irradiation. Representative immunohistochemistry of the calluses stained with OCN antibody at 7 and 14 days after X-ray irradiation (A). Quantification of OCN protein expression in each group (B). n = 4 rats/group,*p<0.05 *vs.* the control group.

Immunohistochemistry revealed increased numbers of proliferating cells during fracture repair in low-dose (0.5 Gy) irradiated rats. This increase in proliferating cells included periosteal cells, osteoblasts and mesenchymal cells or chondrocytes, which are known to contribute to intramembranous ossification, chondrogenesis and endochondral ossification during fracture repair. On day 7, near the fracture site, the periosteal cells stained by PCNA antibodies in the fibrous layer were increased in the low-dose group compared with the non-irradiated group. In contrast, the high-dose irradiated group displayed significantly decreased numbers of PCNA-positive periosteal cells (Fig. 5A, B). In soft calluses, greater chondrogenesis was observed in the 0.5 Gy group on day 7; small, round, premature chondrocytes and mesenchymal cells were detected near the cartilage tissue. The proliferating premature chondrocytes stained by PCNA antibodies were significantly increased in the low-dose group compared with the non-irradiated group. In contrast, deceased PCNA-positive cells, of which mesenchymal cells were predominant, were observed in the high-dose group (Fig. 5C, D). On day 14, a greater reduction in PCNA-positive cells was detected in each group compared with the early phase. The proliferating chondrocytes in the non-irradiated control were more numerous compared with the irradiated groups (Fig. 5C, D). In addition, vascularization was observed in the fibrous calluses, and fewer PCNA-positive mesenchymal cells were noted in the low-dose and control groups. Furthermore, the majority cells in the soft callus area differed in appearance from the hypertrophic chondrocytes observed in the high-dose group, and fibrous tissue was observed in a criss-cross pattern in the cartilage calluses on day 14 (Fig. 5C). In addition, immunohistochemistry demonstrated that low-dose irradiation also increased the expression of OCN protein among proliferating cells on day 14 after irradiation (Fig. 6A, B). PCNA is a protein involved in the major DNA replication repair machinery of cells. When not engaged in DNA replication, PCNA (most often under the control of p53) commits cells to cell cycle arrest and repair of DNA damage. Alternatively, when repair is not possible, absent or low levels of functional PCNA may drive cells to apoptosis [24]. On the one hand, the suppression of PCNA signaling promotes cellular apoptosis [25]. In contrast, PCNA activation suppresses osteoblast apoptosis during *in vitro* culture [26]. These facts reveal that PCNA is also related to cellular apoptosis. Our results indicate that low-dose irradiation increased PCNA protein expression in the calluses. To some degree, this that low-dose irradiation protects cells against apoptosis during fracture repair.

### Osteoblast differentiation was accelerated in the fractures of low-dose irradiated rats

**Figure 7:**
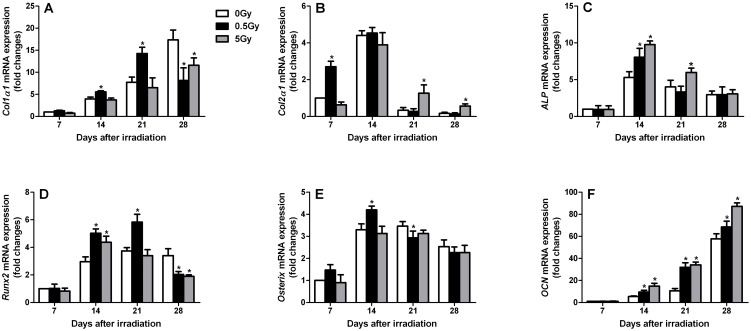
Irradiation-stimulated changes in mRNA expression patterns of osteoblast differentiation-related genes in fracture callus tissues. Real-time PCR was performed at various time points after X-ray irradiation. The following primer sets were used: Col1a1 (A), Col2a1 (B), ALP (C), Runx2 (D), Osterix (E) and OCN (F). n = 4 rats/group,*p<0.05 *vs.* the control group.

The expression of genes involved in osteoblast differentiation was also examined in the callus tissue of fractures from irradiated and non-irradiated rats (Fig. 7). Col2a1 expression was elevated earlier in fractures from low-dose irradiated rats compared with non-irradiated control rats. This observation was consistent with the increase in cartilage observed in low-dose irradiated rats by histomorphometry at day 7, signifying the acceleration of the endochondral phase of fracture repair (Fig. 7B). Moreover, Col2a1 expression was absent in low-dose irradiated and non-irradiated rats by day 21, whereas Col2a1 expression persisted until day 28 in high-dose irradiated rats. Additionally, the expression of the osteoblast-specific transcription factors Runx2 and Osterix was elevated in fracture calluses from low-dose irradiated rats, whereas Runx2 and Osterix expression in the fracture calluses appeared to be not effected by high-dose irradiation (Fig. 7D, E). Furthermore, the expression of the osteoblast differentiation markers ALP and OCN was elevated earlier in fractures from irradiated rats (both low- and high-dose), which is consistent with accelerated osteoblast differentiation (Fig. 7C, F). In the fractures from low-dose irradiated rats, Col1a1 expression appeared earlier, and peak levels were observed by days 14–21 compared with the maximal expression observed at day 28 in non-irradiated control rats (Fig. 7A). ALP expression in fractures from irradiated rats was significantly increased compared with non-irradiated rats on day 14 (Fig. 7C). Furthermore, OCN expression levels were also elevated earlier in fractures from irradiated rats compared with non-irradiated rats, and this increase persisted to 28 days post-irradiation (Fig. 7F). These findings suggest that osteoblasts undergo more rapid differentiation in low-dose irradiated rats; in contrast, high-dose irradiation results in unnatural osteoblast differentiation that is characterized by delayed collagen expression and collagen maturation. Furthermore, this type of rapid osteoblast differentiation in low-dose irradiated rats is consistent with the more rapid completion of endochondral and intramembranous ossification.

### Low-dose irradiation accelerates callus remodeling via enhanced osteoclast-induced signals

**Figure 8:**
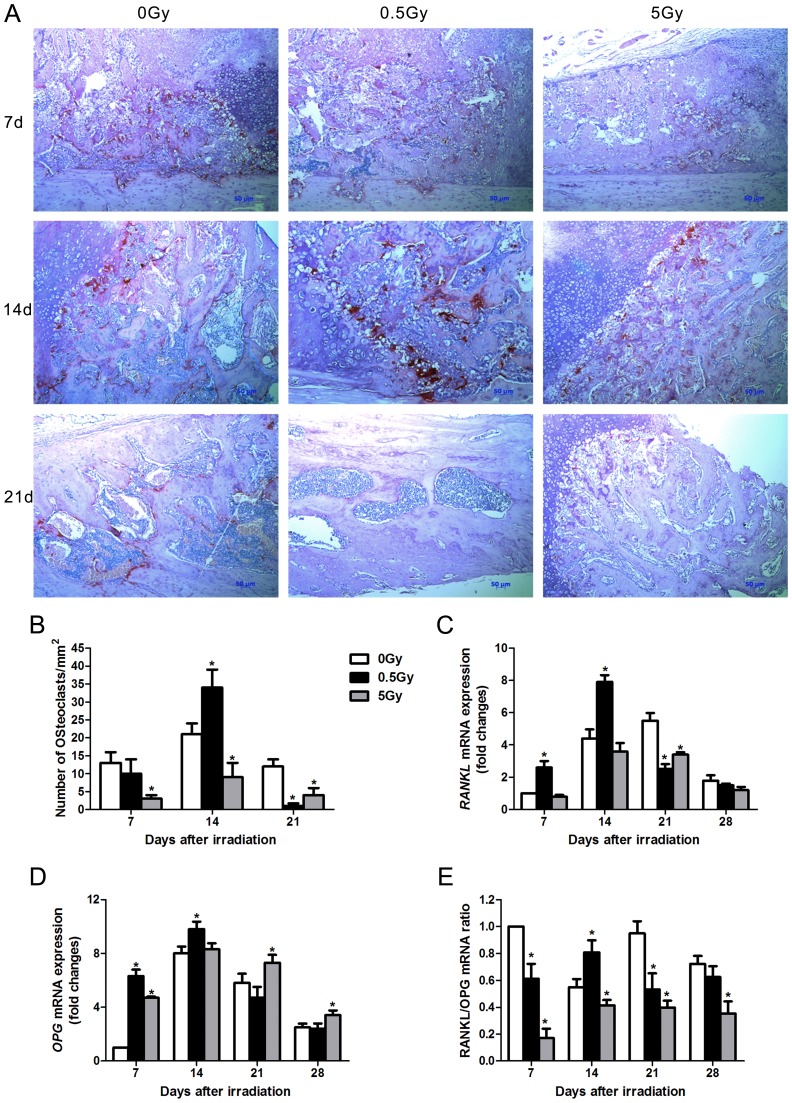
Low-dose X-ray irradiation accelerated bone remodeling. TRAP staining was performed on callus sections at 7, 14 and 21 days after X-ray irradiation (A). Osteoclast numbers were counted in sections per group (B). RANKL and OPG mRNA levels in the fracture callus were examined by real-time PCR (C, D).The RANKL/OPG ratio was calculated (E). n = 4 rats/group,*p<0.05 *vs.* the control group.

The fracture calluses were stained for TRAP expression, which is an osteoclast marker. In day 14, 0.5 Gy X-ray irradiation increased the number of osteoclasts in the fracture calluses by 61.9% (p<0.01) compared with non-irradiated fractures. This observation was consistent with accelerated bone remodeling. By day 21, fractures in the non-irradiated rats contained a certain number of osteoclasts. Surprisingly, few osteoclasts were observed in 21 day-old fractures from 0.5 Gy X-ray irradiated rats. However, high-dose (5 Gy) X-ray irradiation decreased osteoclast numbers at each time-point (Fig. 8A, B). Real-time PCR was performed to measure the expression of the osteoclast-inducing gene RANKL and its receptor antagonist OPG. The fracture calluses from 0.5 Gy irradiated rats exhibited an increased overall expression of both RANKL and OPG, and both genes were elevated earlier in fractures in 0.5 Gy irradiated rats compared with non-irradiated rats (Fig. 8C, D). The maximum expression levels of RANKL occurred at 14 days in the fracture tissue from the 0.5 Gy X-ray group and at 21 days in the control. RANKL expression exhibited a sharp peak in facture tissues from the 0.5 Gy X-ray group, whereas the control group exhibited a lower order of magnitude of expression that was more sustained. The RANKL/OPG radio in 0.5 Gy group was significantly increased compared with non-irradiated group at 14 days. However, the ratio of RANKL/OPG was reduced during fracture repair in the 5 Gy irradiated rats (Fig. 8E). These data were consistent with the differences observed in osteoclast numbers in the fracture calluses. Thus, our results suggest that 0.5 Gy irradiation accelerates callus remodeling by enhanced osteoclast-induced signals.

## Discussion

In this study, we observed increased proliferation and differentiation among osteoblasts in cell culture and fracture healing models stimulated by low-dose irradiation. Given that osteoblasts exhibit a developmental sequence of events, including distinct proliferative, differentiating and mineralizing stages, we selected different time points for the proliferation and differentiation analyses in our study. After single-dose 0.5 Gy X-ray irradiation, osteoblastic cell activity was enhanced, consistent with increased osteoblast proliferation. Similarly, increased numbers of PCNA-positive cells were detected in calluses early during the fracture-healing process. In addition, low-dose (0.5 Gy) X-ray irradiation promoted osteoblastic cell survival and reduced apoptotic cell death based on the sub-G1 cell cycle analysis of cultured cells. Furthermore, low-dose irradiation induced accelerated osteoblast differentiation and fracture healing by triggering the expression of osteoblast-specific differentiation markers and inhibiting apoptosis. These results demonstrated the stimulatory effect of low-dose irradiation on osteogenesis, which was mediated through increased osteoblast proliferation and differentiation.

Despite accumulating evidence highlighting the biological effects of irradiation on bone, a series of controversies remain in this field. For example, although previous researchers have demonstrated the detrimental effects of irradiation on bone [7], [27]–[29], other reports demonstated positive effects of irradiation on osteoblastic differentiation and bone-specific gene expression [16], [18], [20], [30]. Furthermore, a few studies have reported stimulatory effects of low-dose X-ray irradiation (<0.2 Gy) on the proliferation of BM-MSCs, which are osteoblast progenitors and may undergo osteoblastic differentiation in osteogenic medium [16]. These contradictory conclusions may be due to differences in cell lines, irradiation doses and time points used in the gene expression analyses. Our study primarily focused on the effects of low-dose X-ray irradiation on osteoblast proliferation and differentiation both *in vivo* and *in vitro*. Given that the radiosensitivity of cells differs according to cell type and differentiation stage, the relatively low dose of 0.5 Gy was used in our experiments. After 0.5 Gy irradiation, Osteoblast proliferation increased for increased BrdU incorporation and PCNA-positive cells were detected *in vivo* and *in vitro*. The consistent inhibitory effect of high-dose (5 Gy) irradiation on osteoblast proliferation was also reported in this study. A variety of types of DNA damage, including single-strand breaks, double-strand breaks and base damage, occur in response to radiation, and the DNA repair process is activated to address such damage. However, high-dose radiation can induce extensive DNA damage that is difficult to repair and might interfere with cell cycle progression, at which point cells undergo programmed cell death [16], [17], [31]. Adaptive protection against DNA damage and bystander effects might also occur in response to low-dose radiation, generally in terms of the protective mechanisms associated with hormesis [11], [15], [32], [33]. Our results indicate that low-dose irradiation elicited DNA synthesis in osteoblastic cells with an increase in proliferation activity and apoptosis suppression. Although the effect of 0.5 Gy irradiation on osteoblast viability and proliferation was mild, the differences between the control and 0.5 Gy groups were statistically significant. At a minimum, this result indicates that osteoblasts can complete DNA repair at the early stage in response to 0.5 Gy irradiation and that osteoblast proliferation is not inhibited. As demonstrated in our previous study, ≤1.0 Gy irradiation had no significant effect on cell proliferation, viability and apoptosis [20]. This contradiction was attributed to the modulation of the irradiation procedure. In the previous experiment, osteoblasts were irradiated the day after seeding. However, in the present study, we performed an irradiation schedule whereby the irradiated osteoblasts in the logarithmic phase of growth were immediately seeded onto a plate. It is known that different cell types and stages of differentiation exhibit varying susceptibility and responses to radiation exposure.

The osteoblast differentiation process is tightly controlled by bone-specific regulatory factors. Col1 is the main structural component of bone matrix. ALP is important in stabilizing the matrix. OCN is another non-collagenous protein that is almost exclusively expressed in bone and is upregulated during matrix mineralization [34], [35]. In the case of bone, the dynamic and well-regulated expression patterns of these marker genes depend on the differentiation stage of the osteoblasts [18], [35]. For example, Col1 is expressed from the beginning of osteoblast differentiation, whereas only mature osteoblasts in the matrix calcification stage secrete OCN. From our experiments, X-ray irradiation induced significant differentiation effects on osteoblasts. For example, after relatively low-dose irradiation, Col1, ALP and OCN expression was increased in the mature stage of osteoblast differentiation. Similarly, high-dose irradiation also enhanced the expression of osteoblastic differentiation markers with the exception of Col1, which was clearly decreased at all time points. Interestingly, a temporary decline in the expression of several differentiation markers at 4 days after both low-dose and high-dose irradiation *in vitro* was noted, and the following reasons might contribute to this dynamic tendency. First, this decline in expression might represent a cellular survival strategy when radiation damage is present [17]. The major trigger of the cellular response to irradiation is the destruction of genome integrity, and cells can mount a coordinated response to genotoxic stresses, including irradiation, by activating a network of interacting signaling pathways collectively known as the DNA damage response [32]. In response to irradiation, osteoblasts immediately initiate DNA repair mechanisms. Lau et al. [17] reported that discrete γ-H2AX foci that reflect DNA damage were detected as early as 30 min after irradiation in osteoblasts, and approximately all double-strand breaks were effectively rejoined after a repair period of 24 h. As a survival strategy, osteoblasts either retain their proliferative capability and repair DNA damage or are programmed to die (or become senescent or prematurely terminally differentiated) if DNA is heavily damaged [17], [32], [33]. Therefore, cells primarily focus on DNA repair during the early stages after irradiation, whereas the differentiation potential is inhibited. Second, the progressive development of the osteoblastic phenotype, which requires precise coordination between cellular proliferation and differentiation, might play a critical role in the early stages after irradiation [17]. Cell fate is decided according to the balance between self-renewal and differentiation, where cell self-renewal blocks differentiation and supports cell division [36]. Our results revealed that low-dose irradiation stimulated pre-osteoblast proliferation, which may be related to the decreased expression of differentiation markers in the early stage after irradiation. Consistently, following low-dose irradiation, the expression of the transcription factors Runx2 and Osterix, which are key regulators in osteoblast differentiation, was significantly high-dose irradiation. In other words, the increased proliferation observed following low-dose irradiation may constrain the differentiation potentiality in the early stages after irradiation. Although high-dose irradiation increased terminal differentiation among osteoblasts, it inhibited cell proliferation and reduced production of the extracellular matrix. Thus, the cells' bone forming capacity was weakened in response to high-dose irradiation. However, low-dose irradiation enhanced osteoblast proliferation in the early stages, increased the expression of osteogenic markers and promoted the terminal differentiation process. This result demonstrates that low-dose X-ray increases the osteogenic potential of osteoblast.

Fracture healing is a complex process during which mesenchymal stem cells are recruited to the site of injury and subsequently undergo proliferation and differentiation into bone-forming cells [37]. The present study reveals that low-dose irradiation acts as a positive factor in the fracture-healing process given that it promoted cell proliferation, differentiation and fracture healing in animal models. Accelerated intramembranous bone formation and endochondral bone formation were observed in low-dose irradiated fractures. Low-dose irradiation also accelerated callus remodeling by enhanced osteoclast-induced signals. Furthermore, low-dose irradiation enhanced the expression of osteoblast differentiation genes in the fracture calluses. Our *in vivo* experimental results were consistent with the results of osteoblast culture *in vitro*. For example, the number of proliferating cells increased in calluses from fractures irradiated with low-dose X-ray radiation, and gene expression studies from fracture callus tissues demonstrated an enhanced rate of chondrocyte and osteoblast differentiation after low-dose irradiation. Maximal expression of Col2a1 occurred early after low-dose irradiation, demonstrating the acceleration of complete endochondral ossification. These mature, calcified cartilage tissues may act as a template for bone formation and remodeling. Consistent with the acceleration of the cartilage phase of fracture repair, the expression of osteogenic genes, such as ALP, OCN, Col1a1, Runx2 and Osterix, peaked early or were increased in the low-dose irradiated callus tissues. These results document that low-dose X-ray irradiation enhances the bone formation potential of osteoblast in *vitro* and *vivo*. In addition to enhanced bone formation, our findings also suggest that callus remodeling was accelerated upon low-dose irradiation. We performed TRAP staining to determine whether low-dose X-ray irradiation-induced callus remolding was associated with the potential for accelerated osteoclast formation. Our study reveals that the calluses from the rats irradiated by low-dose X-ray irradiation had more osteoclastogenesis. The RANKL/OPG ratio was consistently increased in the fractures after low-dose X-ray radiation. Finally, fracture union was rapid, and the rate of remodeling was enhanced in low-dose irradiated fractures. Our findings are consistent with previous descriptions indicating that low-dose X-ray irradiation promotes the mineralization of fracture callus tissue in rats [19]. However, callus formation and fracture union were delayed in high-dose X-ray-irradiated fractures, and greater collagenous fiber senescence occurred in high-dose irradiated fracture callus tissue. The following reasons contributed to this observation. On one hand, the discoordination between reduced proliferation and abnormal expression of osteogenic genes might result from decreased bone formation. On the other hand, the reduced number of osteoclasts affected callus remodeling, leading to bone matrix aging or sclerosis.

One limitation of this study was that over-active cell proliferation and tumorigenicity should be addressed for all low-dose irradiation interventions. In fact, because current hospital medical imaging procedures may increase patient exposure to low-dose radiation, the risk of cancer development represents a serious concern. Additionally, human epidemiological data warn that irradiation might trigger abnormal cell proliferation and thus promote tumor growth [10]. Nevertheless, our data show that low-dose X-ray irradiation may induce middle and late osteoblast differentiation simultaneously. Another limitation was that we exclusively focused on the effects of radiation on the fracture healing process using a rat fracture model *in vivo* in the present study. However, we failed to detect changes in the distinct regions or non-fractured sites. Irradiation affects bone tissues by both cellular and physicochemical processes, including bone metabolism and fracture healing. The altered compositions of bone tissues lead to differential response to irradiation [38]. Different local environments and components were noted in the fracture or non-fractured sites. In other words, irradiation had different effects on distinct regions of bone. Given the lack of investigations focused on non-fractured bones, the generalization of our conclusion should be interpreted with caution. In addition, low-dose irradiation appeared to accelerate age-related changes in skeletal microarchitecture [39]. In this study, limited passages of osteoblasts and adult animals were employed for *in vitro* and *in vivo* studies. However, it remains unclear whether the stimulatory effects of low-dose irradiation on osteoblasts and fracture healing represent an age-related phenomenon.

## Conclusion

In summary, our experiments revealed that X-ray irradiation at low- or high-doses exhibited opposing effects on osteogenesis. Low-dose (0.5 Gy) X-ray irradiation presented certain beneficial effects on osteoblast proliferation, differentiation and fracture repair. Although high-dose (5 Gy) X-ray irradiation increased terminal differentiation among osteoblasts, it weakened the bone formation capacity of osteoblasts by inhibiting cell proliferation and reducing extracellular matrix production. These findings provide a better understanding of low-dose irradiation-induced biological responses during bone formation and might lead to the development of improved strategies with translational applications for bone-related diseases in humans.
